# The Impact of Physical Activity on Suicide Attempt in Children: A Systematic Review

**DOI:** 10.3390/children12070890

**Published:** 2025-07-06

**Authors:** Marissa Patel, Grace Branjerdporn, Sabine Woerwag-Mehta

**Affiliations:** 1 Faculty of Medicine, Bond University, Robina, QLD 4226, Australia; 2 Mental Health and Specialist Services, Gold Coast Health, Gold Coast, QLD 4215, Australia

**Keywords:** physical activity, suicide attempt, children, team sport

## Abstract

Suicide in children is a major global health crisis, with profound impacts on families, friends, and society. Understanding ways to ameliorate the rate of suicide attempt (SA) is critical given that it is a key factor in predicting future suicide risk. SA is the deliberate act of causing physical injury to oneself with the intent of death. The incidence of SA may be influenced by physical activity (PA). PA includes bodily movement via skeletal muscles that results in energy expenditure and physical fitness. While there is evidence to suggest that PA improves dysregulation of the parasympathetic nervous system which underpins the physiology of suicidal behaviour, evaluating the impact of PA on SA in children is required. **Objectives**: This systematic review aims to determine the relationship between PA and SA in children to inform alternative preventative and interventional strategies. **Methods**: This systematic review was registered with PROSPERO: CRD42023389415. Eight electronic databases were systematically searched. References were transferred to Covidence software for title and abstract screening and full text review were performed based on eligibility criteria: (1) children aged 6–18 years old; (2) participated in PA (individual, group exercise, or team sports); and (3) examined SA as a dependent variable. The JBI Checklist was used to measure the quality and level of bias of included studies. **Results**: Of the 2322 studies identified, 21 were included in the final analysis of the review. Twenty studies were cross-sectional in design, and one implemented a prospective study design. Thirteen studies (61.9%) yielded statistically significant results, indicating that increased PA, particularly team sport, may be associated with reduced odds of SA. There was some evidence to suggest that certain intensities and frequencies of PA may be beneficial to some and detrimental to other subgroups. **Conclusions**: The results suggest that PA may reduce the risk of suicide attempts. Although PA may be associated with reduced SA in children, future research is required, which (1) uses standardised outcome variables; (2) adopts longitudinal and experimental study designs; (3) explores qualitative research to determine distinctive factors that influence participation in PA not captured by quantitative research; and (4) examines different target populations such as children with a broad range of mental health issues.

## 1. Introduction

Suicide is a growing public health concern with close to 800,000 people of all ages dying by suicide each year (one person every 40 s), with children being a highly vulnerable population [1]. Reducing suicide rates remains highly relevant, with the United Nations setting a Sustainable Development Goal of a one-third reduction in suicide mortality rate by 2030 [1]. While risk factors for suicide are well-established [2], a greater understanding of possible preventative strategies may reduce overall prevalence of suicide. Physical activity (PA) appears to be a promising intervention against suicide attempt in adults, and it positively impacts mental health outcomes such as depression and anxiety [3]. However, the impact of PA on SA in children has not been examined. In this review, we aimed to explore the relationship between PA and SA to inform alternative preventive and interventional strategies for suicidal behaviours in children.

### 1.1. Suicidality in Children

Globally, following the COVID-19 epidemic, suicide rates are increasing and represent the fourth leading cause of death in young people aged 15–19 years [1]. The number of deaths by suicide were relatively similar between males and females in this age group representing approximately 40,000 deaths by suicide annually, with the true prevalence likely to be higher due to under reporting [1,4,5]. The economic burden of suicide in children is estimated to be US 500 million annually in Western countries such as Australia [6]. In order to reach the one-third reduction set out by the United Nations, countries need to triple their efforts, increasing the annual average reduction in suicide rates from 1% to 3% [1]. For this systematic review, all children and adolescents aged 18 years and under will be referred to as ‘children’ as per Australian legal definitions [7].

### 1.2. Predictors of Suicide

Suicide attempt (SA) is the strongest predictor of subsequent SA and death by suicide [8]. While SA can occur in many forms (e.g., overdose, hanging, jumping from a height), SA is defined as the process of causing self-harm with the intent to die [9]. The most vulnerable timeframe for completed suicide is within 12 months following SA and during the post-hospital discharge period [10,11]. Female children are almost three times more likely to attempt suicide compared to male children [12]. However, males are three times more likely to die by suicide compared to females [13].

### 1.3. Biopsychosocial Model of Suicide

The aetiology of suicidal behaviour in children can be understood through the biopsychosocial model of suicide that encompasses biological, psychosocial, environmental, and event factors that influence psychological processes [14]. Biological factors that increase the risk of suicide include, genetic predisposition, dysregulation of the hypothalamic–pituitary–adrenal axis affecting serotonin uptake by 5HT receptors, and increased impulsivity associated with a developing brain [15,16,17]. In particular, the physiological mechanism that underpins SA is the dysregulation of the sympathetic and parasympathetic nervous system, with suicidal individuals having decreased parasympathetic nervous system regulation [18]. Psychosocial factors include relationship difficulties, distress or anxiety, with mental illness identified as the leading risk factor for suicide [2]. Environmental influences include trauma, abuse, or history of SA [19]. Event factors represent devastating and significant life events that can cause an individual to attempt suicide based on a perceived inability to cope with a situation, such as loss of a relationship [20]. Protective biopsychosocial factors can also decrease the risk of suicide. There are some protective factors that are well-established, such as self-esteem and resilience having developed in childhood [21,22]. One protective factor that may ameliorate the risk of SA and which is growing its body of evidence is PA. Engagement in PA may reduce the risk of SA as it facilitates favourable biological and psychosocial aspects of a child. In particular, regular PA has been shown to increase parasympathetic dominance through adaptation of the nervous system (i.e., biological) [23]. Furthermore, engagement in regular PA has been associated with a reduction in stress, anxiety and depression as a mechanism of emotional regulation (i.e., psychological), as well as improved social relationships and social inclusion (i.e., social), which are also associated with a lower rate of SA in children [24]. While there are various mediating factors that may contribute to suicidality, this is beyond the scope of the present systematic review.

### 1.4. PA Definitions

PA is defined as bodily movement via skeletal muscles that results in energy expenditure and improves physical fitness [25]. PA can broadly be classified as exercise and non-exercise. Exercise refers to PA that is planned and structured (exercise activity thermogenesis) and non-exercise refers to lifestyle activities embedded into everyday tasks such as walking and cycling to school (non-activity exercise thermogenesis) [25,26,27]. PA can be performed at different intensities including vigorous, moderate and low. Low-intensity PA does not cause a noticeable change in breathing rate and can be sustained for at least 60 min [28]. Moderate-intensity PA can be conducted whilst maintaining a conversation uninterrupted and lasts between 30 and 60 min. Vigorous-intensity PA causes hard breathing and sweating and may last up to 30 min [28].

The types of PA may be generally classified as endurance, strength and resistance training, and flexibility [29]. Endurance PA improves cardiovascular and aerobic fitness through increasing breathing and heart rate; strength and resistance training improves bone and muscle strength through anaerobic fitness; and flexibility improves muscle and joint range of motion [29]. In addition to the numerous health benefits of PA, there are a variety of options to choose from, including bicycle riding, running, swimming, dancing, basketball, soccer, weight training, yoga, and martial arts. This variety makes it a useful tool, as personal preference can be achieved and participation can be individual, group-based, or team-based. International guidelines recommend that children should complete an average of 60 min per day of moderate–vigorous-intensity PA [30]. However, children may participate in PA for less minutes and on fewer days than recommended.

### 1.5. PA in the Context of Suicidality

PA reduces mental health conditions such as anxiety and depression; however, its efficacy in preventing child SA remains unknown [24]. Overall, PA leads to improved mood through serotonin release and fostering of resilience which facilitates positive mental health outcomes through the regulation of the nervous system [31,32]. Nevertheless, the incorporation of PA into routine is essential for healthy physical, social, and emotional development [33]. Children spend the majority of their time at school; subsequently, this setting represents a valuable opportunity to implement PA. PA may also have different effects on SA based on the sex, gender, and ethnicity of the child due to the differences in societal expectations on males and females.

### 1.6. Gap in the Literature

To the author’s knowledge, no systematic reviews have evaluated the relationship of PA on SA in children. Previous reviews investigated SA in adults and explored suicidal ideation and other general mental health outcomes in relation to PA. For instance, Fabiano [34] found a significant decrease in SA in adults following the implementation of PA in a recent systematic review and meta-analysis of randomised control trials. Another systematic review and meta-analysis by Vancomfort [35] found inconclusive evidence regarding the association between PA and suicidal ideation in children. The impact of COVID-19 has been explored in an umbrella review, which found that PA in the form of regular exercise was associated with improved mental health but did not evaluate a causal relationship [36]. Finally, Boelens [37] determined the impact of organised activities on the mental health of children. The authors concluded that PA, in the form of team sports or non-sport activities, improved mental health outcomes, including anxiety and depression.

PA could potentially provide an effective and non-stigmatising adjunct to conventional approaches to suicide prevention and treatment in the adult population given the established association with reduced risk of SA. While PA is associated with improved mental health outcomes in children, little is known about the protective role of PA and suicidality in children. Given that PA appears to be a promising intervention to reduce SA in adults, perhaps the same association exists within the child population. To the best of our knowledge, this systematic review is the first to examine the association between PA and its impact on SA in children. It is hypothesised that PA will reduce the risk of SA in children.

## 2. Methods

### 2.1. Procedure

This systematic review protocol was published on PROSPERO, the international prospective register of systematic reviews on 23 January 2023: CRD42023389415. As this is a systematic review of the published literature, no ethical approval was required. The search strategy including children, PA, and SA (see Table 1) was applied on 19 December 2022 to eight high-quality databases yielding the following results for screening: PubMed (*n* = 953), CINAHL (*n* = 332), EMBASE (*n* = 336), Medline (*n* = 674), ProQuest (*n* = 83), PsycInfo (*n* = 598), Scopus (*n* = 1328), and Web of Science (*n* = 470). The search period was between the inception of databases and 19 December 2022. 

The references were imported into Endnote, and duplicates were removed. The de-duplicated references were transferred to Covidence software (an online platform for systematic reviews that does not utilise version numbers but rather continuously updates software). The articles were screened based on their title and abstract by one author (MP). The full texts were reviewed for inclusion by two independent reviewers (MP and GB). The reviewers met to discuss any discrepancies, with any further disagreements mediated by a third reviewer (SW).

### 2.2. Eligibility Criteria

The inclusion criteria encompassed articles than examined children aged 6–18 years. Studies were included if PA participation (individual exercise, group exercise, or team sports, and of any intensity) was the independent variable, and SA was the dependent variable.

Studies were excluded if they were published in a language other than English. Review articles, including meta-analyses, case reports, case series, conference abstracts, dissertations, expert opinion, letters, and the grey literature, were also excluded. Studies that examined PA and SA as individual outcomes rather than exploring the relationship between both variables were excluded. Furthermore, studies that analysed PA with other health behaviours (e.g., sleep and diet) were also excluded. Studies that focused on self-harm and non-suicidal self-injury (NSSI), sedentary behaviour, high-level athletes and pathological levels of exercise dependence were also excluded (see Table 2).

### 2.3. Data Extraction

Relevant data from eligible studies was extracted using Covidence. Data extraction included the following (where reported/applicable): (i) publication details (first author’s family name, title, and year of publication); (ii) study characteristics (primary objective, type of research design, study duration, sample size, and geographical location); (iii) participant characteristics (age range, method of recruitment); (iv) SA characteristics (timeframe, number); (v) PA characteristics (type, duration, intensity); (vi) study results and conclusions; (vii) limitations; and (viii) clinical implications.

Study characteristics are provided (see Table 3), and the impact of PA on SA is summarised in a narrative synthesis. Other study features, such as participant, PA, and SA characteristics, are also provided. Where possible, quantitative comparisons were made on the information extracted to determine the effectiveness of PA in preventing child SA. Statistical significance was set to a *p* value < 0.05 or a 95% confidence interval. Meta-analysis could not be conducted due to the heterogeneity of the interventions.

### 2.4. Methodological Quality Assessment

Two reviewers independently assessed each article for quality and risk of bias using the Joanna Briggs Institute checklists for cross-sectional studies and cohort studies. These checklists assess risks associated with sampling, measurement, confounding, statistical analysis, and reporting. The items were scored on a scale of 0 (high risk of bias), 0.5 (unclear or potential risk of bias), or 1 (low risk of bias), and no cut-off scores for exclusion were applied to the individual item scores or the overall scores. Not-applicable items were left blank. A mean of items score was calculated to give a final rating of quality (Table 3).

## 3. Results

### 3.1. Study Selection

**Figure 1:**
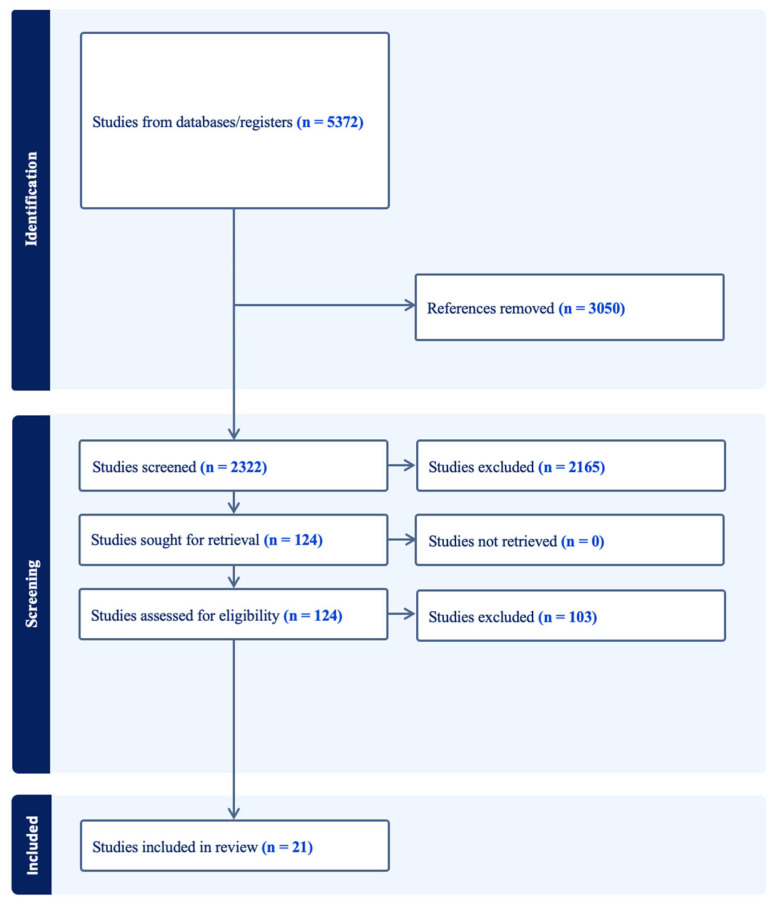
PRISMA diagram providing an overview of the number of included studies from identification, abstract and title screening, full-text review, and inclusion.

The search yielded 5372 articles for screening, with 3050 duplicate references removed. In total, 2322 studies underwent title and abstract screening, 124 full texts were reviewed based on the inclusion and exclusion criteria, and 21 studies were included in data extraction, quality appraisal, and synthesis (see Figure 1). In total, 103 studies were excluded for the following reasons: PA and SA were analysed separately or as a part of cumulative behaviours (*n* = 47, 45.6%); inappropriate study design, such as PhD dissertations or book chapters (*n* = 24, 23.3%); irrelevant outcome measures, for example, sedentary behaviour (*n* = 20, 19.6%); and inability to access study or language other than English (*n* = 11, 10.7%). Of note, one study by Cho [56] was excluded at the full-text review stage due to its unresolvable differences in methodology and results.

### 3.2. Demographics and Study Design

About half of the studies were conducted in the United States of America (*n* = 12, 57.1%). Other studies also sampled only one country, including Korea (*n* = 3, 14.3%), China (*n* = 1, 4.8%), and Sierra Leone (*n* = 1, 4.8%). Four studies sampled multiple countries [31,39,49,50], with two set in countries of varied socio-economic status [31,50], one set in low- and middle-income countries [49], and one set in low-income countries only [39]. The age range of the participants was between 11 years and 18 years of age, indicative of children in junior high school and senior high school. Participation was voluntary, and surveys were administered in a school setting based on attendance. The sample sizes ranged from 628 to 276,169 children. Eleven studies (52.4%) had 10,000–100,000 participants. Other studies varied from less than 1000 participants (*n* = 3, 14.3%), 1000–10,000 participants (*n* = 3, 14.3%), and greater than 100,000 participants (*n* = 4, 19%) (see Table 3). The mean sample size was 50,230 participants, with a standard deviation of 67,615. Twenty studies (95.2%) were cross-sectional, and one study [46] used a longitudinal study design.

### 3.3. Independent Variable (PA)

The way PA was defined varied across studies. Nine studies (42.6%) reported the broad category of PA without specifying type (e.g., individual, group, aerobic), twelve studies (57.1%) used team sport, and two studies (9.5%) outlined specific types of PA (biking, walking, muscle strengthening). The duration of PA was quantified in nine studies (42.6%) but not specified in the remaining studies. Approximately half of the studies (*n* = 12, 57.1%) reported the number of days exercised each week. Of these studies, nine (42.9%) reported answer options as dichotomous and three (14.3%) required participants to exercise on all seven days of the week. The intensity of exercise was calculated in six studies (28.6%).

### 3.4. Dependent Variable (SA)

The authors utilised a variation in the following question as an outcome measure for SA: “During the past 12 months, how many times did you actually attempt suicide?”. This question is widely used in the literature and can be seen in standardised tools such as the Youth Risk Behaviour Survey (YRBS) and Global School-Based Student Health Survey (GSHS). In total, 61.9% of studies (*n* = 13) adopted questions from the YRBS, 23.8% (*n* = 5) of studies implemented the GSHS, and 14.3% (*n* = 3) studies created their own outcome measure or did not specify the primary outcome measure used. One study further examined SA that resulted in injury, poisoning, or overdose that required treatment by a doctor or nurse [53].

### 3.5. Increase in PA Reduces SA (Inverse Relationship)

Thirteen studies (61.9%) yielded statistically significant inverse associations, indicating that increased PA reduced the odds of SA in children (see Table 4). Of the remaining 8 studies, most demonstrated statistically non-significant results but still showed an inverse trend (*n* = 7, 87.5%).

### 3.6. Increase in PA Increases SA (Direct Relationship)

One study (4.8%) by Lee [55] showed a direct association where male and female children who engaged in moderate or vigorous PA demonstrated an increased risk of SA. Mixed results were found for seven studies (31.2%). Two studies (14.3%) that reported an inverse association also showed that risk of SA increased based on gender and intensity. Similarly, five studies (62.5%), which demonstrated non-significant inverse associations, also revealed that PA significantly increased the likelihood of SA due to gender and intensity of exercise.

### 3.7. Quality Assessment

The studies were assessed using JBI quality assessment checklists. Nearly all studies presented low risk relating to sample selection. Independent and dependent variables were measured using validated or best practice measures in all studies. The largest risk of bias related to confounding variables. Nine studies failed to mention, or only briefly mentioned, covariates that may bias results and did not adequately control for these confounders. Four studies did not use the most appropriate or robust analytic techniques to achieve their aims or failed to adequately describe all analytical methods.

## 4. Discussion

### 4.1. General Findings

This is the first systematic review to assess the impact of PA on SA in children. We aimed to identify if there was an inverse relationship between PA including types, levels and frequency of PA, and SA. Overall, there was an association in support of our hypothesis that regular PA and, in particular, team sport was protective against SA. However, only two studies (9.5%) outlined specific types of PA, including biking, walking, and muscle strengthening. Almost half of the included studies (42.6%) reported the broad category of PA without specifying their type. Given this limited data, a thorough examination of the impact of specific types of PA on SA in children was unable to be performed. Rather, a detailed exploration of the potential association between PA as a broad category and that certain frequencies and intensities of PA may be beneficial for some and detrimental to other subgroups will be discussed below.

### 4.2. Intensity of PA

Southerland [45] demonstrated that regular vigorous PA for more than 20 min a day on three or more days per week reduced odds of SA. Contrary to traditional recommendations of regular daily moderate–vigorous PA for 60 min, short intensive sessions of PA appeared protective against SA. Tao [54] also revealed non-significant findings in support of low, moderate or vigorous-intensity PA reducing the likelihood of SA. In this study, the authors formulated their own survey questions and perhaps compromised statistical significance with a lack of standardised outcome measures. The health benefits of being physically active can be understood through improved quality of life (QOL) and psychological wellbeing [57].

A clear association emerged between intensity of PA and reduced SA based on gender. Brown [38] established that SA decreased when male participants were engaged in vigorous PA on six or more days per week. Similarly, Felez-Nobrega [31] concluded that male children across 48 countries had reduced SA when engaged in moderate–vigorous-intensity PA for 60 min daily. In addition, Brown [38] concluded that females who participated in vigorous-intensity PA on 3–5 days per week were protected against SA. Regular vigorous-intensity PA appears protective for both males and females at different frequencies. Individuals who engage in PA are more likely to be aware of the benefits of PA and a healthy lifestyle, including improved sleep, which has also been shown to reduce the risk of suicide [58].

One study, published in Korea, revealed any intensity of PA increased the likelihood of SA. Lee [55] found that regular moderate and frequent vigorous-intensity PA increased the risk of SA for males after adjusting for body image (perceived body weight compared to friends). When considering females, regular and frequent vigorous-intensity exercise resulted in an increased risk of SA also after adjusting for body image concerns. These findings differ from the findings of Kim [59], who showed that Korean children who were underweight or who perceived themselves as overweight had an increased risk of SA. Additionally, perhaps these findings can be explained through an over representation of suicide attempters given that the leading cause of death among children in Korea is suicide [60].

Overall, regular vigorous-intensity PA for more than 20 min a day on three or more days per week appeared to reduce the odds of SA in children of Western countries such as the USA [54]. Although vigorous intensity can be defined as hard breathing and sweating, the type of PA to achieve this remains undetermined. Regarding gender, males may be afforded the protective benefit of PA on SA when engaging in daily moderate–vigorous-intensity PA for 60 min [31,58]. Whereas females may be protected when engaging in the same intensity and duration of PA on 3–5 days per week [58]. However, children in Korea may not be afforded the same protective benefit of moderate–vigorous-intensity PA [45].

### 4.3. Team Sport and SA in White/Western/High Income Countries

Nine studies examined the impact of team sport participation on SA. Taliaferro [46] was the only study with a prospective design allowing for analysis of causal relationship between team sport participation and SA. The authors conducted a 5-year case–control, longitudinal study that demonstrated team sport participation during middle school and high school lowered rates of SA compared to non-participants. Discontinuing sport after middle school resulted in a 2.38 times increased risk of SA in high school when controlling for demographics, moderate–vigorous PA, depressive symptoms, and previous SA in middle school [46]. Youth who discontinue sport appear to lose the protective social networks that team sport provides. Equally, students who only participate in team sport in high school are not afforded the same benefits of team sport on reducing SA as those who commenced in middle school. This important finding can be explained by the nature of team sport progressively becoming more high level and competitive with age which may negatively impact self-efficacy of students who lack skills or financial resources to participate.

Brown [38], who analysed from the 2003 YBRS, showed that both males and females benefited from participating in a team sport. Lester [42] revealed that participation in one or more team sports was associated with reduced SA (7.6%) compared to non-participants (9.8%) over a 20 year period from 1991 to 2011 when analysing results from 11 YRBS timepoints. Taliaferro [47] also analysed results of YRBS administered every two years from 1999 to 2007. Southerland [45], who showed reduced odds of SA independent of confounding factors such as screen time, drug abuse, and weight misperception, utilised data from the 2010 Tennessee Middle School YRBS. These results suggest that the benefits of team sport participation on reduced SA may operate independently of the listed confounding factors. However, the overlap in data utilised in these studies mean these data should be interpreted as a whole rather than as independent analyses.

Harrison [40] and Page [44] analysed team sport participation in the context of school which included physical education (PE) or extra-curricular activity. Harrison [40] demonstrated that team sport participation for 1–2 h or more per week decreased odds of SA. Similarly, Page [44] found that the relative risk of both male and female students participating in one or two team sports corresponded to reduced odds of SA.

While 77.8% of studies that analysed team sport participation yielded statistically significant results, two studies (22.2%) revealed non-significant results. Sabo [53] found that team sport tended to be protective against SA regardless of gender, despite not reaching statistical significance. West [13] produced mixed results. Firstly, the authors reported non-significant findings to suggest that team sport participation may be protective against SA in female children. Secondly, they demonstrated statistically significant results suggesting a 1.52 times increased likelihood of SA for male adolescents when participating in team sport. However, the overall low response rate of 68% does not provide an accurate representation of the population and the voluntary nature of participation may have impacted the findings through self-selection bias.

Overall, team sport participation encourages social integration that is protective against SA, which may be an advantage over individual sport [53]. Positive social relationships and peer encouragement established with teammates, coaches, and the wider sporting community have clear protective benefits. Specifically, the participants are afforded opportunities to learn how to effectively overcome setbacks in a supported way that fosters a sense of resilience. Given that all studies investigating team sport participation were conducted in a first world Western country, the results can be generalised to other Western countries.

### 4.4. Active School Travel

Active school travel (AST) is classified as lifestyle based non-exercise and represents an important aspect of PA [27]. Chen [39] and Arat [49] both evaluated the impact of AST in the form of walking or biking. Chen [39] determined that walking or biking to school in 34 low-income countries decreased the odds of SA by 18% regardless of gender. Arat [49] produced mixed results. Walking or biking to school appeared to reduce SA for children in China, Philippines, Pakistan, and Thailand despite not reaching statistical significance. The decision to walk or bike to school is influenced by many factors not accounted for including distance to school, safety of infrastructure, and individual socioeconomic status and could have influenced the results.

Walking or biking to school in Sri Lanka increased the rate of suicide by 1.45 times compared to children who did not engage in this type of PA [49]. The recent rise in suicidality in Sri Lanka may be explained through contextual factors such as low socio-economic status and community alcohol misuse, independent of an individual’s personal attributes [61]. This could be a reflection of collective trauma experienced during almost three decades of civil war resulting in displacement of children from their families and unsafe environments affected by conflict-related violence in the form of landmines and communal massacres. The fundamental impacts on families and individuals remains largely unknown given that the civil war only ended recently in 2009 [62]. Perhaps in certain low- and middle-income countries, where access to AST is hindered by other contextual factors, the benefit of incidental exercise is not seen.

### 4.5. School Sport Participation

Schools provide increased opportunities to address the positive impact of PA on mental health. Southerland [45] showed that PE attendance two–five times per week reduced likelihood of SA. These results were significant regardless of whether students participated in individual or team based PA. Oler [43] demonstrated that participation in school athletics (interschool team sport) was protective against SA for females. Logistic regression revealed that athletic participation reduced risk of SA by 2.7 times compared to non-participants (*p* = 0.02). The positive impact of PA in this case may be understood through sports participation providing an opportunity for development of identity and fostering of personal and social resources. This self-development is protective against risk taking behaviours such as SA [53].

Hu [50] produced mixed results based on 71 varied income countries which can be explained through low class attendance. Attending PE class for two, three, or four days per week was significantly associated with an increased risk of SA. Students who attended PE class four days per week represented 3% of all students, those who attended for three days represented 4.8%, and those who attended for two days represented 19.2% of all students. The reason for low attendance is unstated and results may underrepresent children who experience poor mental health outcomes that could benefit from PA but who do not attend school. The results were inconclusive, as the authors also found an inverse association between PE attendance for one and five days per week. Interestingly, higher rates of attendance were seen on these days (35.9% and 17.2%, respectively), suggesting that results may have reached significance level with a larger and more accurate representation of the child population.

The relationship between sport participation (individual or team-based) and reduced SA may be understood through cultural resource theory that encapsulates two phenomena: resource building and gender identity [53]. Resource building refers to increased social integration and status attainment as protective against suicidality. When applied to a sporting context, PA can be character-building, allowing for the development of friendships, social acceptance, and leadership skills that contribute to success in adult life [63,64,65]. The impact of culture, the media, and society on gender identity has been institutionalised as ‘masculine’ and ‘feminine’ [53]. The traditional feminine script corresponds to fragility, passivity, and compliance, resulting in an internalisation of difficult issues such as suicidality, which leads to higher rates of SA. Contrastingly, the masculine script dictates externalising problem behaviours and utilising personal attributes to solve the issue [66]. Sport has increasingly been characterised as ‘masculine’, where males and, increasingly, females are encouraged to adopt athletic practices of competitiveness, goal attainment, and assertiveness [67]. Consequently, any tendency towards internalising suicidality may be weakened. The integration of resource building and gender identity in the context of PA fosters attributes protective against SA.

### 4.6. Muscle Strengthening

Muscle strengthening at least three times per week appears to lower the odds of SA across all sexes and genders [6,41]. Further research is needed to establish if muscle strengthening is protective against SA as this systematic review yielded non-significant inverse associations.

### 4.7. The Role of Sex and Gender

PA appears protective against SA for male children. Completing 60 min of moderate–vigorous-intensity PA per day reduced the odds of SA across 48 varied income countries [31]. A combination of PA for at least 20 min, three–seven times per week and team sport participation decreased the odds of SA in male children [48]. The most protective combination was completing PA three–five times per week and team sport participation suggesting that the addition of team sport may reduce the threshold benefit of regular PA to three–five days in the male population.

When lesbian or bisexual females engaged in light PA for 3–4 days per week, the risk of SA was 6.186 times higher compared to female children who completed more than 5 days of light PA [41]. Light PA appears to only be protective against SA for female children when completed on most days of the week, suggesting that intensity and frequency of PA influence how PA protects against SA. The positive impacts of light-intensity PA on mental health and mood is attributed to increasing whole-body blood circulation, which leads to brain stimulation [68]. The positive role of PA on reducing SA was further supported by Jang [41], who demonstrated that lesbian or bisexual females who performed no vigorous PA each week had a 7.152 increased risk of attempted suicide compared to females who performed over 5 days of vigorous PA. The results concerning lesbian, gay or bisexual people should be interpreted with caution given the vulnerability of this group who are already at an increased risk of SA [69].

PA can have negative health implications in certain female subgroups. Felez-Nobrega [31] revealed that females who completed moderate–vigorous-intensity exercise for 60 min per day had an increased risk of SA. Furthermore, females who engaged in PA six–seven times per week and those who participated in a team sport were at a 1.88- and 1.5-times increased risk of SA, respectively. The association between PA and SA may be more complex in female children. Many female children perceive themselves to be overweight and body image concerns may be a motivating factor to participate in intensive PA, as females tend to employ active coping styles in response to stress [70]. Female children may have an underlying predisposition to suicidal behaviours due to stressors associated with body image. High levels of stress are linked to an increased risk of suicide [71]. Consequently, females who are already at a heightened risk of SA, due to body image concerns may engage in PA as a coping strategy which can account for the negative mental health outcome associated with PA that was found in this case [31].

### 4.8. Ethnicity

Lester [42] analysed data from 11 YRBS administered across the USA to determine the impact of ethnicity on PA and SA. Team sport participation reduced the odds of SA for Whites and Hispanics. Taliaferro [47] found that for boys, regardless of ethnicity, sports team participation lowered the risk of SA. For girls, being White compared to other ethnicities, reduced the odds of SA. Interestingly, team sport participation increased the odds of SA for Black girls, Hispanic girls, and Asian girls. This direct association can be explained by sport participation existing within institutional structures influenced by class, gender and race [53]. The increased rates of SA in minority ethnic girls participating in team sport can also be attributed to experiences of marginalisation and bullying, which are identified risk factors for SA [72]. Team sport participation may be a risk factor for ethnic minority girls living in a Western country.

### 4.9. Potential Pathological Aspects of PA

Despite excluding studies that examined exercise dependence, trends demonstrated that a pathological aspect of PA may exist. Arat [49] revealed that exercising for 60 min per day corresponded to a 1.39 times increased risk of SA in the Philippines. Kwon [51] also demonstrated a 1.2 times increased risk of SA for Korean children when exercising for more than 60 min per day on four or more days per week. Excessive exercise may be associated with psychological stress as a result of competition and performance anxiety leading to dysregulation of the autonomic nervous system [23,73].

Additionally, participation in multiple team sports demonstrated increased likelihood of sustaining serious injury following a SA. Sabo [53] found that suicidal male athletes are more likely to incur serious injury compared to boys that did not participate in sport. Male children who participated in 1–2 teams and attempted suicide were approximately three times more likely to result in serious injury and those involved in three or more teams were approximately five times more likely to sustain serious injury requiring medical attention [53]. These results are supported by the concept of hegemonic masculinity suggesting that suicidal boys are more likely to sustain serious injury due to externalisation of behaviours consistent with violence and stoicism [67]. Alternatively, perhaps coaching style in regard to being goal orientated or values driven influences how PA interacts with SA [74]. Overall, boys are more prone to serious injury or death by suicide due to an increased prevalence of impulsiveness and aggressiveness [19].

Although there appears to be a protective benefit of PA on reduced SA, excessive exercise or participation in team sport may represent a pathological aspect of PA. Two studies highlighted that exercising for 60 min per day may correspond to an increased risk of SA. Specifically, Sabo [55] suggests that male children who participate in multiple team sports are more prone to serious injury after SA. Further understanding of this important nuance will assist in understanding how different types or contexts of PA may have differential effects on mental health and suicidality.

### 4.10. Clinical Implications

Based on the findings of this review PA appears to be a cost-effective adjunct in suicide prevention for children. The protective aspects of PA include improved mood, positive social relationships, and higher levels of resilience leading to reduced SA. We were unable to determine a dose–response relationship between level of PA and SA. However, regular PA appeared to reduce odds of SA. Female children tended to benefit from light-moderate vigorous-intensity PA on 3–5 days per week whereas, male children appeared to benefit from moderate–vigorous-intensity PA on 6–7 days per week. Team sport also provided promising findings supporting reduced SA in children despite several confounding factors such as type of team sport and socio-economic status, that may impact an individual’s ability to participate in team sports. Team sport provides an effective preventative strategy against SA, as participants can elect a team sport that suits their personality, interests, and PA level, while still enjoying the protective benefits. It is recommended that the findings of this systematic review encourage future longitudinal experimental research with the aim of developing public health programmes tailored to the individual child and group setting. The implementation of high-quality, evidence-based sport programmes would afford children the possible protective benefit of physical activity when utilised in the correct setting.

The complex interaction between PA and SA cannot be underestimated. Indeed, our findings suggest that a pathological aspect of exercise may exist. Moderate–vigorous-intensity exercise on all days of the week was detrimental for some female children [31,55]. Furthermore, the statistically significant benefit of PA on reduced SA was lost when adjusting for feeling sad or hopeless amongst male and female children [38].

In the context of public health and school-based interventions, the provision of PA opportunities may be a potentially beneficial suicide prevention strategy. This may be through school-based classes, interschool sport, extra-curricular club-based sport, and training before and after school, as well as by ensuring that these sports are easily accessible and a low cost to parents. Having AST infrastructure such as good bicycle paths, concrete footpaths and public transport options may also promote this PA when commuting. Children need access to a wide variety of PA choices, such as dancing, tennis, swimming, martial arts, soccer, baseball, or basketball to choose what they like, develop competence, build their self-esteem, and develop their sense of achievement. Encouraging movement compared to winning and competitiveness may also support children to be willing to try new PAs and develop skills in this way.

### 4.11. Limitations of Included Studies

Despite the numerous benefits of this systematic review, as discussed above, there are several limitations to consider. The primary limitation is the potential overlap of population data analysed in the included studies. Eighteen of the twenty-one studies used data from publicly available national surveys: the YRBS, implemented by the Centers for Disease Control (CDC) in the United States of America [75]; the Korean YRBS, collected by the Korea CDC [76]; and the Global School-Based Student Health Survey (GSHS), developed by the World Health Organization [77]. Seven of these studies utilise data from overlapping years [13,38,42,44,47,48,53], meaning that many of the results presented in this study are based on duplicate data. Secondly, most studies were cross-sectional in design, preventing the establishment of a cause–effect relationship. Despite most results suggesting an inverse association, we are unable to confirm if PA reduced SA or if children pre-disposed to suicide naturally engaged in PA. Thirdly, the data used was self-reported. The subjective nature of surveys created potential for reporting bias whereby participants may have responded in a manner that was socially acceptable but perhaps not a true reflection of their engagement with PA. Fourthly, due to the lack of interventional studies, we were unable to determine the most beneficial type of PA or the differences between team and individual sport to protect against SA as many studies included examples of exercise in answer options such as basketball, soccer, running, or swimming laps. Lastly, the scope of the systematic review did not examine the mediating effect of variables such as mental health conditions, overweight/obesity, or low socioeconomic status, which may plausibly affect the association between PA and SA.

### 4.12. Recommendations for Research

Most studies (95.2%) included in this review were cross-sectional in nature, preventing establishment of a causal relationship. Although evidence suggests a protective benefit of increased PA on reduced risk of SA in children in certain circumstances, further research is required in order to recommend PA to reduce risk of SA in children. Future research would benefit from longitudinal and experimental/interventional study designs with PA as the intervention, particularly utilising randomised controlled trial designs, as they allow for establishment of cause–effect relationships. Clinically relevant subgroup analysis and consistent standardised measures across all outcome variables should be utilised to facilitate comparability between studies, such as through a meta-analysis. Specifically, subgroup analysis would be based on gender, risk factors, children with a broad range of mental health issues, and the most protective type of PA. Future high-quality methodological studies may allow for PA to be implemented as a treatment strategy for suicidality. In this case, PA would be a cost-effective alternative way to talk therapy, which children often do not engage in. Additionally, a qualitative approach may be useful to elicit distinctive factors that influence participation in PA not captured by quantitative research such as quality of relationships among individuals in team sports.

## 5. Conclusions

This is the first systematic review that determines the impact of PA on SA in children. Overall, team sport appears to be protective against SA in children, and the school setting provides an increased opportunity to incorporate PA into a child’s routine through individual and team sports. Team sport, however, may not be protective against SA for certain female ethnic minority groups. Short regular intensive sessions of vigorous PA appear effective across both sexes. For male children, the risk of SA was reduced when engaged in daily moderate–vigorous-intensity PA for 60 min, whereas female children benefited from a reduced frequency and shorter duration of sessions on three to five days per week. However, exercise may be detrimental for males who engage in three or more team sports, and females who engage in high volume daily moderate–vigorous-intensity PA. AST also appeared to lower the risk of SA across many countries. Although PA appears to be a useful strategy to prevent SA when not used in excess and in the correct setting, a causal relationship cannot be established given that most included studies were cross-sectional in nature. Therefore, we are unable to recommend PA as a strategy to reduce SA in children despite the promising results.

## Figures and Tables

**Table 1:** Search strategy based on population, intervention, and outcome.

Search Strategy		
Population	Children and Youth	Child * Youth Adolescen * “Young people” Teenager * Paediatric Paediatric Young person
Intervention	Physical activity	Exercise “Physical activity” Sport “Sedentary activity” “Sedentary behaviour” “Sedentary lifestyle”
Outcome	Suicide	Suicid * “Suicidal attempt” “Acute suicidality” “Chronic suicidality” “Suicide attempts”

((Exercise) OR (Physical activity) OR (Sport) OR (Sedentary activity) OR (Sedentary behaviour) OR (Sedentary lifestyle)) AND ((Child *) OR (Youth) OR (Adolescen *) OR (Young people) OR (Teenager *) OR (Paediatric) OR (Paediatric) OR (Young person)) AND ((Suicid *) OR (Suicidal thoughts) OR (Suicidal behaviour) OR (Suicidal attempt) OR (Suicidal behaviour) OR (Suicidal ideation) OR (Suicidal plan) OR (Acute suicidality) OR (Chronic suicidality) OR (Non-Suicidal Self Injury) OR (Suicide attempts)). “Comparator” within PICO has been omitted given that any comparator was included.

**Table 2:** Inclusion and exclusion criteria for included studies based on the PICO format.

	Inclusion Criteria	Exclusion Criteria
Population	Children and adolescents aged 6–18 years	High level athletes
Intervention	Physical activity or exercise participation including individual exercise, group exercise or team sports as the independent variable	- Physical activity examined with other health behaviours (e.g., sleep and diet) - Sedentary behaviour - Pathological levels of exercise dependence
Comparison	Any comparator	
Outcome	Suicide attempt as the dependent variable	- Self-harm and non-suicidal self-injury (NSSI) Relationship between physical activity and suicide attempt not examined and only considered as individual variables
Other methodological considerations		- Non-English articles - Review articles including meta-analyses Case reports, conference abstracts, dissertations, expert opinion, letters, and the grey literature

**Table 3:** Demographics (country, age group, sample size), parameters of physical activity, including type, duration per day, number of days, and intensity, and the main results for the included studies (N = 21) examining the relationship between physical activity and suicide attempts.

Author and Year	Demographics	Dataset Used	Type of Physical Activity				Results	Quality Assessment
			Type	Duration per Day	Number of Days	Intensity		
		Statistically Significant Results ↑ Physical Activity ↓ Suicide Attempts (*n* = 14)						
Brown, 2007 [38]	USA 14–18 years N = 10,530	YRBS, 2003	Team sport and participation in PA (non-specified)	Vigorous = at least 20 min of exercise that resulted in sweat and breathing hard Moderate = at least 30 min of exercise that did not result in sweat or breathing hard	Vigorous PA Frequent ≥ 6 days Regular vigorous 3–5 d Moderate PA ≥ 5 d Insufficient = moderate PA 1–4 d	Moderate or vigorous	Sports team participation lowered odds of SA in both boys and girls Engaging in vigorous PA (regular participation for girls and frequent for boys) lowered the OR of SA When participants engaged in both types of PA or felt sad/hopeless associations were not statistically significant (NSS)	86%
Chen, 2021 [39]	34 low-income countries 13–17 years N = 127,097	GSHS, 2010–2017	Walking or biking to school	Dependent on distance to school	Active school travel ≥5 d	Not specified (NS)	Children with active school travel had lowered OR of SA (country wide)	100%
Felez-Nobrega, 2020 [31]	48 varied income countries 12–15 years N = 136,857	GSHS, 2009–2016	Sports, country-specific PA	Meeting WHO PA guidelines = At least 60 min	Moderate–vigorous PA on 7 d/w	Moderate and vigorous	Meeting PA guidelines reduced OR of SA in boy and female children	100%
Harrison, 2003 [40]	USA 14–15 years N = 50,168	Minnesota Student Survey, 2001	Team sport participation at school	NS	1–2 h/w	NS	School team sport participation for 1–2 h/week reduced odds of SA	64%
Jang, 2017 [41]	Korea 12–18 years N = 628	Korean YRBS, 2015	PA (non-specified) Muscle strengthening exercises	>10 min O >20 min	1–2 days 3–4 days >5 days	Low intensity = walking Vigorous PA = breathing hard or sweating	Regular daily PA in the form of light- or vigorous-intensity exercise appears protective against suicide attempt for lesbian or bisexual girls	100%
Lester, 2017 [42]	USA 13–18 years N = 138,690	YRBS, 1991–2011	Team sport	NS	NS	NS	Team sport participation was associated with fewer suicide attempts for both male and female children, Whites and Hispanics.	57%
Li, 2021 [6]	USA 14–18 years N = 13,677	YRBS, 2019	Team sports (≥one team)	NS	Muscle strengthening ≥3 times/w	NS	Participation in team sport was associated with a lower risk of SA	100%
Oler, 1994 [43]	USA 14–18 years N = 823	NA	School team sport participation	NS	NS	NS	Team sport participation at school reduced SA for female children. OR for SA was higher in non-athletes compared to athletes	100%
Page, 1998 [44]	USA 13–18 years N = 12,272	YRBS, 1991	Team sport participation at school	NS	NS	NS	Students participating in one or two team sports were significantly less likely to have attempted suicide (girl 1.3 times less likely and boys 1.47 times less likely)	64%
Southerland, 2016 [45]	USA 11–14 years N = 60,715	Tennessee Middle School YBRS, 2010	PA (NS) Team sport School sport participation (PE)	At least 20 min	PA < 3 days/week = infrequent PA ≥3 days/week = regular	NS	Regular PA on ≥3 days/week, team sport participation and PE attendance all independently reduced odds of suicide attempt	100%
Taliaferro, 2011 * [46]	USA 14–18 years N = 71,854	YRBS, 1999–2007	Team sport	NS	NS	NS	Participating in team sport throughout middle and high school was protective independent of confounding factors Discontinuing team sport after middle school increases OR of SA in high school	94%
Taliaferro, 2010 [47]	USA 11–18 years N = 739	NA	Team sport	NS	NS	NS	Team sport participation in boys and girls reduced OR of SA	79%
Unger, 1997 [48]	USA 12–18 years N = 10,506	YRBS, 1991	PA (non specified exercise or sports activities) Team sport	≥20 min of PA	1–2 times/week 3–5 times/week 6–7 times/week	Vigorous PA	PA and team sport participation is protective against suicide attempt for male children The most protective combination is completing physical activity 3–5 times per week and playing on a team sport	71%
		Non Statistically Significant Results ↑ Physical Activity ↓ Suicide Attempts (*n* = 9)						
Arat, 2017 [49]	China, Phillipines, Indonesia, Sri Lanka, Thailand, Pakistan 11–17 years N = 23,372	GSHS, 2003–2009	Walk/bicycle PA (NS)	PA ≥ 60 min	7 d/w	NS	Walking or biking to school correlated with a reduction in SA in China, Philippines, Pakistan and Thailand PA for 60 min/d correlated with a reduction in SA in children (China and Indonesia)	86%
Hu, 2022 [50]	71 varied income counties 11–18 years N = 276,169	GSHS, NS	School sport participation	NS	1–2 d ≥3 d	NS	Attending physical education for 1 day only or for 5 days or more each week was associated with lower odds of suicide attempt	93%
Jang, 2017 [41]	Korea 12–18 years N = 628	Korean YRBS, 2015	PA (non-specified) Muscle strengthening exercises	>10 min OR >20 min	1–2 d 3–4 d >5 d	Low intensity = walking Vigorous PA = breathing hard or sweating	Vigorous PA or muscle strengthening exercises for 1–4 d/w may reduce SA in gay or bisexual boys Light PA or muscle strengthening exercises for 1–4 days p/w may reduce SA in lesbian or bisexual girls	100%
Kwon, 2022 [51]	Korea 12–18 years N = 7498	Korean YRBS, 2019	PA (NS)	>60 min	1–3 d 4–7 d	NS	PA for 60 min/d for 1–3 d/w may reduce SA	79%
Li, 2021 [6]	USA 14–18 years N = 13,677	YRBS, 2019	Muscle strengthening exercises	Not specified	≥3/w	NS	Muscle strengthening exercise for ≥3 times/w may reduce SA	100%
Peltzer, 2021 [52]	Sierra Leone 14–17 years N = 2798	Sierra Leone GSHS, 2017	PA (NS)	≥60 min	7 d/w	NS	Daily PA ≥60 min/d may reduce SA	86%
Sabo, 2005 [53]	USA 14–18 years N = 16,262	YRBS, 1997	Team sport	NS	NS	Moderately involved: 1–2 teams Highly involved: ≥3 teams	Team sport participation reduced SA in male and female children Rates of injury were higher in athletes who engaged in SA and team sport compared to boys who engaged in SA and did not participate in team sport	100%
Tao, 2007 [54]	China 12–18 years N = 5453	NA	PA (NS)	Vigorous = activity making you sweat and breathe hard for ≥20 min or 7.5 metabolic equivalents (METS) Moderate = activity making you not sweat or breathe hard for ≥20 min or 4 METS	Low = PA 200–400 METS, 2–3 session of moderate or vigorous PA Moderate = PA ≥400–<560 METS, e.g., 5 session moderate PA or 3 session of vigorous PA High = PA ≥ 560, e.g., daily moderate PA or ≥4 session vigorous PA	Low-moderate and vigorous	Low-moderate and high engagement in PA may reduce SA	100%
West, 2020 [13]	USA 12–18 years N = 14,041	YRBS, 2007	Team sport	NS	NS	NS	Team sport participation may reduce multiple SA in female children	64%
		Statistically Significant Results ↑ Physical Activity ↑ Suicide Attempts (*n* = 7)						
Arat, 2017 [49]	China, Phillipines, Indonesia, Sri Lanka, Thailand, Pakistan 11–17 years N = 23,372	GSHS, 2003–2009	PA (NS) Walk/bicycle	PA ≥ 60 min	7 d/w	NS	PA for 60 min/d increased SA (Philippines) Walking/biking to school increased OR for SA (Sri Lanka)	86%
Felez-Nobrega, 2020 [31]	48 varied income countries 12–15 years N = 136,857	GSHS, 2009–2016	Walking to school and PA (country-specific examples)	Meeting WHO PA guidelines = ≥60 min	Moderate–vigorous PA on 7 d/w	Moderate and vigorous	Meeting PA guidelines were associated with higher OR of SA in girls	100%
Hu, 2022 [50]	71 varied income counties 11–18 years N = 276,169	GSHS, NS	School sport participation	NS	1–2 d ≥3 d	NS	Attending PE class for 2, 3 or 4 d/w was associated higher OR of SA in girls	93%
Kwon, 2022 [51]	Korea 12–18 years N = 7498	Korean YRBS, 2019	PA (NS)	>60 min	1–3 d 4–7 day	NS	PA 4–7 times/w increased OR of SA	79%
Lee, 2013 [55]	Korea 12–18 years N = 74,698	Korean YRBS, 2007	PA (NS)	Moderate = ≥30 min PA that did not make them sweat or breathe hard Vigorous = ≥20 min PA that made them sweat or breathe hard	Moderate Insufficient 1–4 d Regular ≥5 d Vigorous Insufficient 1–2 d Regular 3–4 d Frequent ≥5 d	Moderate and vigorous PA	Male children who engaged in regular moderate PA had an increased likelihood of suicide attempt when controlling for body image Female children who performed any amount of vigorous PA were at an increased of suicide attempt compared to girls who performed no vigorous PA when controlling for body image, stress, and depression	100%
Unger, 1997 [48]	USA 12–18 years N = 10,506	YRBS, 1991	PA alone (NS exercise or sports activities) Or in combination with team sport	≥20 min of PA	1–2 times/w 3–5 times/w 6–7 times/w	Vigorous PA	Female children who engaged in PA 6–7 times per week and participated in team sport had increased OR of SA	71%
West, 2020 [13]	USA 12–18 years N = 14,041	YRBS, 2007	Team sport	NS	NS	NS	Team sport participation increased OR for SA in boys	64%

Notes: ↑ = Increase in physical activity; ↓ = Decrease in suicide attempt; * The only longitudinal study included in this review. GSHS = The Global School-based Health Survey; YRBS = Youth Risk Behaviour Survey; /w = per week; d = day; NS = not specified; N = total sample size; NSS = not statistically significant; OR = odds ratio; CI = confidence interval; *p* = *p*-value; SA = suicide attempt; PA = physical activity.

**Table 4:** The direction of relationship and statistical significance between physical activity and suicide attempts based on intensity, team sport, active school travel, school sport, muscle strengthening, sex and gender, ethnicity, and potential pathological aspect of physical activity.

Theme	Author	Statistically Significant Results (Inverse Association) ↑ Physical Activity ↓ Suicide Attempts	Non-Statistically Significant Results (Inverse Association) ↑ Physical Activity ↓ Suicide Attempts	Statistically Significant Results (Direct Association) ↑ Physical Activity ↑ Suicide Attempts
Intensity	Southerland, 2016 [45]	Regular vigorous PA for more than 20 min a day 3 or more days per week reduced odds of SA (OR: 0.93, 95% CI: 0.87–0.99).		
	Brown, 2007 [38]	Vigorous PA six or more days per week decreased SA for boys (OR = 0.44, 95% CI = 0.21–0.96). Vigorous-intensity PA 3–5 days per week was protective against SA for girls (OR = 0.67; 95% CI = 0.45–0.99).		
	Felez-Nobreg, 2020 [31]	60 min of daily moderate–vigorous-intensity PA reduced SA for male children across 48 countries (OR = 0.78; 95% CI = 0.70–0.86).		
	Lee, 2013 [55]			Regular moderate (OR = 1.37; 95% CI = 1.13–1.67) and frequent vigorous-intensity (OR = 1.22 95% CI = 1.00–1.48) PA increased the risk of SA for boys after adjusting for body. Regular (OR = 1.23; 95% CI = 1.07–1.41) and frequent-vigorous-intensity (OR = 1.41; 95% CI = 1.16–1.71) exercise resulted in an increased risk of SA for girls also after adjusting for body image. Frequent vigorous-intensity remained significant for increasing odds of suicide attempt in both male (OR = 1.23; 95% CI = 1.00–1.50) and female children (OR = 1.30; 95% CI = 1.06–1.60) when further adjusting for stress and depression.
Team Sport	Taliaferro, 2011 [46]	Team sport participation during middle school and high school lowered rates of SA compared to non-participants (4.5% vs. 7.4%; *p* = 0.006). Discontinuing sport after middle school resulted in a 2.38 times increased risk of SA in high school (95% CI = 1.04–5.41).		
	Brown, 2007 [38]	Both boys (OR = 0.5; 95% CI = 0.33–0.76) and girls (OR = 0.66; 95% CI = 0.53–0.82) benefited from participating in a team sport.		
	Lester, 2017 [42]	Participation in one or more team sports was associated with reduced SA (7.6%) compared to non-participants (9.8%) (*p* < 0.001).		
	Taliaferro, 2010 [47]	Participation in one or more team sports resulted in fewer suicide attempts for boys (OR = 0.62; 95% CI = 0.53–0.74) and girls (OR = 0.79; 95% CI = 0.07–0.09).		
	Southerland, 2016 [45]	Team sport participation may reduce the odds of SA independent of screen time, drug abuse and weight misperception (OR: 0.70; 95% CI: 0.65–0.75).		
	Harrison, 2003 [40]	Team sport participation for 1–2 h or more per week decreased odds of SA (OR = 0.45; 95% CI = 0.39–0.52).		
	Page, 1998 [44]	Relative risk of both boy (95% CI = 1.08–2.01) and girl (95% CI = 1.06–1.59) students participating in one or two team sports corresponded to reduced odds of SA.		
	Sabo, 2005 [53]		Team sports appeared to be protective against SA for both girl (OR = 0.86; 95% CI = 0.65–1.14) and boy (OR = 0.83–95% CI = 0.52–1.32) children. Female children who participated in 1–2 team sports (OR = 0.92; 95% CI = 0.65–1.25) and 3 or more team sports (OR = 0.73; 95% CI = 0.52–1.02) appeared to reduce risk of SA.Male children who participated in 1–2 team sports (OR = 0.93; 95% CI = 0.54–1.58) and 3 or more teams (OR = 0.70; 95% CI = 0.45–1.08) also appeared to be protected against SA.	
	Tao, 2007 [54]		Low-moderate engagement in PA (OR = 0.61; 95% CI = 0.34–1.09) and high engagement in PA (OR = 0.95; 95% CI = 0.52–1.76) appeared protective against SA.	
	West, 2010 [13]		Team sport appeared protective against SA for female children (OR = 0.89; 95% CI = 0.70–1.14). Team sport may also be protective against injuries sustained following SA in female children (OR = 0.69; 95% CI = 0.38–1.26).	Male children participating in team sport had an increased risk of SA (OR = 1.52; 95% CI = 1.07–2.16).
Active School Travel	Chen, 2021 [39]	Walking or biking to school in 34 low-income countries decreased the odds of SA by 18% regardless of gender (OR = 0.82; 95% CI = 0.75–0.90).		
	Arat, 2017 [49]		Walking or biking to school appeared to reduce SA for children in China (OR = 0.75; 95% CI = 0.48–1.18), Philippines (OR = 0.98; 95% CI = 0.834–1.13), Pakistan (OR = 0.87; 95% CI = 0.68–1.12) and Thailand (OR = 0.83; 95%CI = 0.62–1.12). Exercising for 60 min daily also appeared to reduce the risk of SA in China (OR = 0.72; 95%CI = 0.46–1.12) and Indonesia (OR = 0.82; 95% CI = 0.47–1.41).	Walking or biking to school in Sri Lanka increased the rate of suicide by 1.45 times compared to children who did not engage in this type of PA (95% CI = 1.10–1.93).
School Sport	Southerland, 2016 [45]	PE attendance 2–5 times per week reduced likelihood of SA (OR:0.88; CI: 0.82–0.95).		
	Oler, 1994 [43]	Participation in school athletics (interschool team sport) was protective against SA for girls (*p* = 0.04)		
	Hu, 2022 [50]		Attending PE for one (OR = 0.93–95% CI = 0.84–1.04) or five days (OR = 0.89; 95% CI = 0.80–1.00) appeared to reduce risk of SA.	Attending PE class for two (OR = 1.05; 95% CI = 0.94–1.18), three (OR = 1.19; 95% CI = 1.00–1.41) or four (OR = 1.13; 95% CI = 0.93–1.38) days per week was significantly associated with an increased risk of SA.
Muscle Strengthening	Jang, 2017 [41]		For male children, muscle strengthening exercises for 1–2 days (OR = 0.541; 95% CI = 0.196–1.499) or 3–4 days (OR = 0.575; 95% CI = 0.196–1.6787) per week appeared to reduce the likelihood of SA. Similarly, muscle strengthening exercises for female children 1–2 days (OR = 0.893; 95% CI = 0.164–4.852) or 3–4 days (OR = 0.277; 95% CI = 0.044–1.756) also appeared protective against SA.	
	Li, 2021 [6]		Muscle strengthening for at least 3 times per week (OR = 0.74; 95% CI = 0.55–1.01) appeared to be protective against SA.	
Sex and Gender	Felez-Nobreg, 2020 [31]	60 min of moderate–vigorous-intensity PA per day reduced the odds of SA for boys (OR = 0.78; 95% CI = 0.70–0.86).		Girls who completed moderate–vigorous-intensity exercise for 60 min per day had an increased risk of SA (OR = 1.22; 95% CI = 1.10–1.35). Girls who engaged in PA 6–7 times per week and those who additionally participated in a team sport were at a 1.88- and 1.5-times increased risk of SA, respectively (PA 6–7 times per week: OR = 1.88; 1.31–2.70. PA 6–7 times per week and team sport participation: OR = 1.50; 95% CI 1.09–2.05).
	Unger, 1997 [48]	PA for at least 20 min, 3–7 times per week and team sport participation decreased the odds of SA in male children (PA 3–5 times per week: OR = 0.4; 95% CI = 0.23–0.72. PA 6–7 times per week: OR = 0.5; 95% CI = 0.29–0.85). The most protective combination against multiple suicide attempts was completing PA 3–5 times per week and team sport participation (OR = 0.18; 95% CI = 0.08–0.39) followed by PA 6–7 times per week and team sport participation (OR = 0.28; 95% CI = 0.15–0.54).		
	Jang, 2017 [41]	Lesbian or bisexual girls who engaged in light PA for 3–4 days per week had a 6.186 times higher risk of SA compared to female children who completed more than 5 days of light PA (*p* = 0.011). Lesbian or bisexual girls who performed no vigorous PA each week had a 7.152 increased risk of attempted suicide compared to girls who performed over 5 days of vigorous PA (95% CI = 1.273–40.164).	For gay or bisexual boys, vigorous PA 3–4 days per week may be protective against SA (OR = 0.796; 95% CI = 0.24–2.64). For lesbian or bisexual girls, light PA for 1–2 days (OR = 0.127; 95% CI = 0.008–2.041) may be protective against SA.	
Ethnicity	Lester, 2017 [42]	Team sport participation reduced the odds of SA for Whites and Hispanics (*p* = 0.001 for both Whites and Hispanics).		
	Taliaferro, 2010 [47]	Sports team participation lowered the risk of SA for boys regardless of ethnicity (OR: 0.69 CI: 0.58–0.81). For girls, being White, compared other ethnicities, reduced the odds of SA (OR = 0.61 CI: 0.51–0.73).		Team sport participation increased the odds of SA for Black (*p* = 0.001), Hispanic (*p* = 0.01), and Asian girls (*p* = 0.01).
Potential Pathological Aspect of Physical Activty	Arat, 2017 [49]			Exercising for 60 min per day corresponded to a 1.39 times increased risk of SA in the Philippines (95% CI = 1.03–1.86).
	Kwon, 2022 [51]		Exercising for 60 min a day for 1–3 days (OR = 0.968; 95% CI = 0.801–1.171) may be protective against SA.	Exercising for more than 60 min per day on four or more days per week yielded a 1.2 increase in SA (*p* = 0.047).
	Peltzer, 2021 [52]		Exercising for 60 min daily (OR = 0.73; 95% CI = 0.51–1.06) appeared to reduce odds of SA.	
	Sabo, 2005 [53]			Male children who participated in 1–2 teams and attempted suicide were approximately three times more likely to result in serious injury (OR = 2.81; 95% CI = 2.81) and those involved in three or more teams were approximately five times more likely to sustain serious injury requiring medical attention (OR = 4.84; 95% CI = 1.92–12.2).

Notes: ↑ = Increase in physical activity; ↓ = Decrease in suicide attempt; OR = odds ratio; CI = confidence interval; *p* = *p*-value; SA = suicide attempt; PA = physical activity.

## Abbreviations

The following abbreviations are used in this manuscript:

PA	Physical activity
SA	Suicide attempt
NSSI	Non-suicidal self-injury
YRBS	Youth Risk Behaviour Survey
GSHS	Global School-Based Health Survey
PE	Physical education
AST	Active school travel
